# Multimodal fusion image enhancement technique and CFEC-YOLOv7 for underwater target detection algorithm research

**DOI:** 10.3389/fnbot.2025.1616919

**Published:** 2025-06-19

**Authors:** Xiaorong Qiu, Yingzhong Shi

**Affiliations:** School of Internet of Things Engineering, Wuxi Institute of Technology, Wuxi, China

**Keywords:** underwater image, multi-weight fusion, CFEC-YOLOv7, image enhancement, multimodal fusion

## Abstract

The underwater environment is more complex than that on land, resulting in severe static and dynamic blurring in underwater images, reducing the recognition accuracy of underwater targets and failing to meet the needs of underwater environment detection. Firstly, for the static blurring problem, we propose an adaptive color compensation algorithm and an improved MSR algorithm. Secondly, for the problem of dynamic blur, we adopt the Restormer network to eliminate the dynamic blur caused by the combined effects of camera shake, camera out-of-focus and relative motion displacement, etc. then, through qualitative analysis, quantitative analysis and underwater target detection on the enhanced dataset, the feasibility of our underwater enhancement method is verified. Finally, we propose a target recognition network suitable for the complex underwater environment. The local and global information is fused through the CCBC module and the ECLOU loss function to improve the positioning accuracy. The FasterNet module is introduced to reduce redundant computations and parameter counting. The experimental results show that the CFEC-YOLOv7 model and the underwater image enhancement method proposed by us exhibit excellent performance, can better adapt to the underwater target recognition task, and have a good application prospect.

## 1 Introduction

Occupying about 70 per cent of the Earth’s surface, the oceans are an important repository of aquatic and energy resources, and are crucial to the long-term development of human society. As land resources become more and more limited and marine technology advances, the fields of marine resource exploration, fishery management and ecological protection rely more and more on cutting-edge observation technologies and high-performance computing systems for object recognition in underwater images. This process is especially critical for the in-depth understanding of the underwater environment and the automatic avoidance, recognition and accurate positioning of underwater targets, which is also necessary for the autonomous operation of underwater robots. Therefore, improving the accuracy and efficiency of object recognition in underwater images not only promotes the development of marine scientific research, but also provides strong support for the sustainable use and protection of marine resources. Based on this, target recognition technology based on underwater optical images has become one of the important directions in computer vision research. In this process, the application of emerging technologies has laid a solid foundation for solving various challenges encountered in underwater imaging.

Underwater photography is essential for ocean exploration and seabed data collection. However, compared to images taken on land, underwater images are often degraded by water currents, light absorption and scattering, noise, uneven lighting, and camera motion, leading to blur and reduced quality. These issues hinder accurate analysis and target recognition. Therefore, improving image processing techniques to enhance important details, extract useful information, and remove noise is crucial. Enhanced images not only improve visual clarity but also boost the performance of tasks like feature extraction. Developing underwater-specific image enhancement algorithms is therefore of great scientific and practical importance.

In traditional underwater target recognition, signal acquisition, feature extraction, and classification are key steps. Feature extraction, in particular, has been time-consuming and reliant on expert knowledge, limiting the process to semi-automation. With rapid advancements in AI and deep learning, along with improved hardware performance, image recognition accuracy has greatly increased—sometimes even surpassing human levels. As a foundational technology, image recognition plays a vital role in enabling intelligent robots to achieve precise visual localization, navigation, and underwater operations. High-precision image recognition is essential for advancing robotic intelligence. In recent years, deep learning-based image recognition has not only progressed in land applications but also become increasingly important for underwater environments. Researchers are now developing end-to-end, universal image recognition systems for underwater use, aiming to enhance robot perception and efficiency through full automation. This advancement significantly promotes the intelligence and versatility of underwater robots in both civilian and military contexts. Therefore, exploring underwater target recognition methods holds great value in areas such as marine resource management and environmental monitoring, as well as in military reconnaissance and beyond.

## 2 Related work

Due to the special nature of the underwater environment, in the process of acquiring underwater images, it will be affected by the scattering of light, absorption, and turbidity of water quality, which will produce static blurring phenomena such as low contrast and colour distortion in the acquired underwater images, as well as dynamic blurring phenomena caused by changes in the depth of field due to unfocusing of the aperture of the underwater imaging equipment and the displacement of the relative motion generated between the photographed underwater target and the imaging equipment, which seriously affects the clarity and quality of underwater images are seriously affected.

Therefore, in order to solve the problem of static blurring, it is necessary to enhance the underwater images, thereby improving the quality and clarity of the underwater images. In recent years, with the continuous progress of digital image processing technology, many researchers at home and abroad have devoted themselves to extracting the effective information in underwater images in order to obtain clearer images. Ghani (2018), in order to improve the extraction rate of valuable information and contrast in underwater images, proposed an underwater image enhancement algorithm that integrates homomorphic filtering, recursive overlapping CLAHS and dual image wavelet fusion in a staged process algorithm. Hou et al. (2019) proposed a new variational model based on non-local differential operators that incorporates an underwater dark channel prior and a quadtree subdivision method to estimate the transmission map and global background light, and also used a fast algorithm based on the alternating direction method of the multipliers to speed up the solution process in order to solve the problem of blurring and low contrast that occurs in underwater images. Hegde et al. (2020) proposed an adaptive estimation single image enhancement algorithm in CIELAB colour space to remove blurring and restore image colours in underwater images and resulted in good enhancement of the images from the standard underwater coral reef image dataset used. In order to solve the problems of colour distortion and colour bias, Zhuang et al. (2021) studied the Bayesian retinex improvement algorithm based on the Retinex algorithm to enhance various underwater images, and established a mathematical base model for the overall colour correction of underwater images with the help of calculating the reflectivity and illuminance of the recovered underwater image and the original image. Li et al. (2022) established a mathematical base model for the overall colour correction of the underwater images by constructing an adaptive colour and contrast enhancement framework to remove the noise and restore the colour in the image, in which Gaussian differential filter and bilateral filter are used to decompose the high frequency and low frequency components respectively, and then soft thresholding operation is used to suppress the noise in the high frequency component and use adaptive colour and contrast enhancement strategy to enhance the low frequency component, which improves the quality of the underwater image. Zhang et al. (2023) considered the factor of underwater light attenuation and proposed an image enhancement strategy that combines colour correction and multi-scale fusion. With the wide application of deep learning technology in the field of image processing, the research of underwater image enhancement algorithms has been gradually carried out. Ye et al. (2018) investigated the haze detection and colour correction problem of a single underwater image based on a deep learning approach, and proposed a framework based on stacked conditional generation of adversarial networks, which learns the mapping between the underwater image and the image in the natural condition in an end-to-end manner. Lu et al. (2019) proposed an underwater image restoration method based on a multi-scale recurrent generative adversarial network system to convert underwater style images into restoration styles to solve the problem of turbidity and colour distortion caused by the underwater environment. In order to improve the overall quality of underwater images and to solve the blurring problems occurring in underwater images, Yang et al. (2020) continuously enhanced underwater images based on conditional generative adversarial networks to achieve clear underwater colour images with the help of multi-spatial scale generation, and a dual discriminator was used to capture both local semantic picture information and global semantic information. Wang et al. (2021) proposed an underwater image enhancement convolutional neural network (UICE^2-Net) using two-colour space, which implements basic operations, such as denoising and removing colour casts, through RGB pixel-level blocks, and globally adjusts the brightness, colour and saturation of the underwater image using HSV global adjustment blocks and a new neural curve layer, and combines the RGB and HSV blocks by assigning weights to each pixel to output the image merit blocks, which achieves good image restoration results. The method achieves better image recovery results.

To overcome the problem of dynamic blur, blurring and low contrast of underwater images, Xu et al. (2022) firstly designed a novel convolutional neural network to estimate illuminance and obtain reflectance. Based on this, the method changes the traditional retina-based low-light enhancement processing idea, and performs colour balance and illumination correction on the decomposed reflectance and illuminance respectively, and finally produces fused reflectance and illumination images through post-processing to overcome the blurring and blurring problems. Cheng et al. (2023) proposed a new Transformer-based perceptual contrast network for underwater image enhancement by embedding the Transformer into the UIE network in order to solve the limitation of the purely convolution-based network, which is the first time that the contrast learning is applied to the underwater image enhancement task. Zeng et al. (2021) proposed a method to add an adversarial occlusion network to the standard Faster R-CNN algorithm. Using the competition between the adversarial occlusion network and the Faster R-CNN network, the latter learns how to block a given target, making it difficult for the detection network to correctly classify the blocked target, which ultimately results in a better robustness of the recognition network to underwater targets.

To address the issues of improving the accuracy and speed of underwater target detection, Cai et al. (2022) proposed a weakly-supervised learning framework for underwater target recognition based on the simultaneous training of two deep learning detectors and letting them train each other based on the selection of cleaner samples seen during training, achieving a balance between accuracy and speed. Lyu et al. (2023) proposed a YOLOX-based improved detection algorithm, EFP-YOLO, in order to recognise dense, small-sized underwater targets. The algorithm enhances the ability to extract features of the underwater targets and fuses, in a parallel interactive manner, the local and global information, where an asymmetric task-focused head is proposed to improve the scale-aware, spatial-aware and task-aware capabilities of the detection head to achieve accurate counting of marine benthic organisms. Yu et al. (2023) designed an underwater network U-YOLOv7 based on the YOLOv7 network in order to discriminate the diversity and dense distribution of aquatic species for underwater biological detection. Wang et al. (2025) develop a discriminative underwater image enhancement method empowered by large foundation model technology to address the discriminativeness between underwater color disparities in foreground and background regions. Wang et al. (2024) developed an adaptive attenuated channel compensation method based on optimal channel precorrection and a salient absorption map-guided fusion method for eliminating the color deviation in the RGB color space. Wang et al. (2024) developed a reinforcement learning-based human visual perception-driven image enhancement paradigm for underwater scenes. Although these methods can enhance the image contrast and improve the image colour distortion to a certain extent, there are cases of over-enhancement or under-enhancement, which makes the comprehensiveness, stability and robustness of the traditional underwater image enhancement algorithms unsatisfactory. Due to the slow speed of the large network and the huge scale of the model, the above methods are not effective when applied directly to underwater scenes. Underwater scenes are more complex than land scenes, which leads to generally lower image quality acquired by underwater imaging devices, and underwater targets are usually small and dense, which poses a great challenge for recognition. Therefore, we propose a highly accurate and fast underwater image enhancement recognition algorithm. Our main contributions are as follows:

(1) Aiming at the static blurring problem of underwater images, an adaptive color compensation algorithm is proposed to compensate for the light attenuation caused by light scattering and absorption in underwater images. Then, an improved color restoration algorithm based on multi-scale Retinex is proposed to restore the distorted colours. Finally, a multi-weight fusion algorithm is proposed to improve the contrast of underwater images.
(2) For the problem of underwater dynamic blur, the Restormer network is used to train the synthesised underwater dynamic blur images to obtain the pre-training weights. Finally, the pre-training weights are used to remove the dynamic blur from the real underwater dynamic blur images.
(3) Addressing the issues of low target recognition accuracy and slow recognition speed in underwater environments. We proposed the CCBC module and the FasterNet module to fuse local and global feature information in a parallel interactive manner, provide rich shallow image semantic information for advanced deep convolutional features, and make better use of the computing power of the device.

## 3 Multimodal fusion for image enhancement with CFEC-YOLOv7 model

### 3.1 Image enhancement with multimodal fusion

#### 3.1.1 Static blur removal algorithm

**Figure 1:**
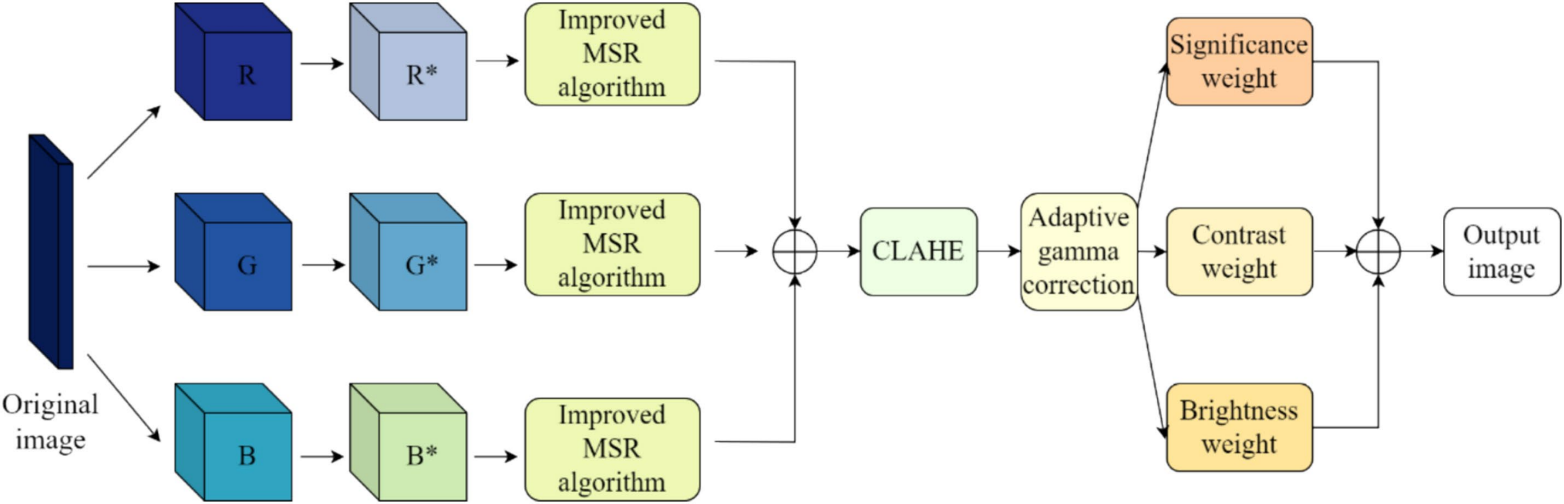
Underwater image de-static blurring flowchart.

Static blur is a blurring phenomenon in underwater images caused by low contrast, colour distortion, uneven illumination and other blurring phenomena due to the absorption and scattering of light by water and its suspended matter during the imaging process. To solve the problem of static blurring in underwater images, we use the corresponding algorithms of colour compensation, colour restoration and contrast enhancement in order to solve the problem of static blurring in underwater images, and the specific flowchart is shown in Figure 1.

#### 3.1.2 Adaptive colour compensation algorithm

The colour distortion in static blurring is mainly due to the difference in water depth which results in different degrees of attenuation due to the absorption and scattering of different coloured light. Therefore, in order to better recover the distorted colours of underwater images, we propose an adaptive colour compensation algorithm, which can adaptively compensate the colours of channels with serious attenuation.

For our proposed adaptive colour compensation algorithm, we first consider the average of the pixel values of each colour channel (*I_R_*, IG, IB) as shown in Equations 1–3:


(1) $${\bar{I}}_{R}=\text{mean}(I_{R}(i,j))$$

(2) $${\bar{I}}_{G}=\text{mean}(I_{G}(i,j))$$

(3) $${\bar{I}}_{B}=\text{mean}(I_{B}(i,j))$$

where ${\bar{I}}_{I},{\bar{I}}_{G},{\bar{I}}_{B}$ , are the average pixel values of the red, green, and blue channels, respectively, and *I_R_*(*i*, *j*), *I_B_*(*i*, *j*) and *I_G_*(*i*, *j*) are the pixel values of the red channel, the green channel, and the blue channel, respectively. Then, the mean values of each colour channel are arranged in the order of mean size from largest to smallest to obtain the maximum value *T_max_*(*i*, *j*), the intermediate value *T_mid_*(*i*, *j*) and the minimum value *T_min_*(*i*, *j*). The compensation coefficients for compensating the intermediate value channel and the minimum value channel are calculated based on the maximum, intermediate, and minimum values, as shown in Equations 4, 5.


(4) $$m=\frac{T_{\max}(i,j)-T_{\min}(i,j)}{T_{\max}(i,j)+T_{\min}(i,j)}$$

(5) $$n=\frac{T_{\max}(i,j)-T_{\mathrm{mid}}(i,j)}{T_{\max}(i,j)+T_{\mathrm{mid}}(i,j)}$$

Where *m* is the compensation coefficient for compensating having the minimum value channel and *n* is the compensation coefficient for compensating having the intermediate value channel.

The compensated having intermediate value channel and having minimum value channel are shown in Equations 6, 7.


(6) $$I_{m\mathrm{id}}(i,j)=T_{\mathrm{mid}}(i,j)+nT_{\max}(i,j)$$

(7) $$I_{\min}(i,j)=T_{\min}(i,j)+mT_{\max}(i,j)$$

In Equation 6, *I_mid_*(*i*, *j*) is the compensated colour channel with intermediate values; in Equation 7 *I_min_*(*i*, *j*) is the compensated colour channel with minimum values. The three channels are combined to give the colour compensated image *I_C_*.

#### 3.1.3 Colour recovery algorithm based on improved MSR

In order to recover the colour of underwater images, we propose the improved MSR algorithm. The original MSR algorithm for colour recovery of underwater images will make the processed underwater images have problems such as colour bias, being over-smoothed, and unclear texture details. Therefore, in order to solve these problems, we improve the MSR algorithm by introducing the rolling bootstrap filtering function, and then use the improved MSR colour recovery algorithm to process the *R*, *G*, and *B* colour channels of the colour compensated image *I_C_* separately. Firstly, according to the scale definition of the underwater image structure, Gaussian filter with appropriate intensity is used to eliminate the small-scale edges and detailed textures, and then the large-scale edge structure is recovered, in order to effectively solve the problem of colour distortion in underwater images and the problem of easy loss of edge details and colour bias when the underwater image is enhanced by the improved MSR algorithm. The roll-guided filter function is defined according to the scale of the underwater image structure as large-scale structure and small-scale structure.

In the first step, a Gaussian filter is used to erase all the required small-scale detail information in the underwater image and a rough estimation of the illumination component is performed as shown in Equations 8, 9.


(8) $$J=\frac{1}{K_{p}}\sum_{q\in N(p)}\exp(-\frac{\|p-q\|2}{2\sigma_{s}^{2}})I_{C}(q)$$

(9) $$K_{p}=\sum_{q\in N(p)}\exp(-\frac{\|p-q\|2}{2\sigma_{s}^{2}})$$

Where, *Kp* is used to normalise the ownership in Gaussian filtering, *p*, *q* denote the coordinates of different pixel points in the image, respectively; *I_C_*(*q*) denotes the grey value of pixel point *q* of the image after the colour compensation process; and *N*(*p*) is the set of domain pixels of pixel point *p*. The standard deviation. *σ_S_* is used to control the window size of the Gaussian filtering kernel, wherever the pixel distance is less than *σ_S_*. The structures are smoothed out, but at the same time the edges of large scale structures are blurred out to some extent.

The second step uses the Gaussian-filtered small-scale structure of the image as a guide image and the colour-compensated image *I_C_* as input for secondary processing by means of bilateral filtering. This processing aims to iteratively recover the blurred large-scale structure and accurately estimate the illumination component. In the next iteration, the bootstrap image is the output image of the previous iteration and the input image is the colour compensated input image. As the number of iterations increases, the progressively clearer large-scale structures are restored and highlighted, protecting edges and enhancing details in the underwater image, resulting in the final image *I*_MSR*_. As shown in Equations 10, 11.


(10) $$J^{t+1}(p)=\frac{1}{K_{p}}\sum_{q\in N(p)}\exp(-\frac{\|p-q\|2}{2\sigma_{s}^{2}}-\frac{J^{t}(p)-J^{t}{\left(q\right)}^{2}}{2\sigma_{r}^{2}})I(q)$$

(11) $$K_{p}=\sum_{q\in N(p)}\exp(-\frac{\|p-q\|2}{2\sigma_{s}^{2}}-\frac{J^{t}(p)-J^{t}{\left(q\right)}^{2}}{2\sigma_{r}^{2}})$$

In Equation 10, *K_p_* is used for normalization, *I*(*q*) is the same as *I_C_*(*q*) in Equation 8 and refers to the colour compensated image as the input image, J*^t^* denotes the last iteration output image, t refers to the number of iterations, and *J^t^*(*p*) or *J^t^*(*q*) refers to the grey value of the pixel point with the coordinates of *p* and *q* in the *J^t^* image. *σ_r_* is used for controlling the weighting of intensity difference (e.g., grey level difference).

Substituting Equation 10 into the original MSR algorithm as in Equation 12 yields the improved MSR algorithm as shown in Equation 13.


(12) $$R_{\mathrm{SSR}}^{c}(x,y)=\log R^{c}(x,y)=\log I^{c}(x,y)-\log[G{\left(x,y\right)}^{*}I^{c}(x,y)]$$

(13) $$R_{\mathrm{MSR}}^{c^{*}}(x,y)=\sum_{n=1}^{N}W_{n}\{\log I^{c^{*}}(x,y)-\log[J^{t+1}{\left(p\right)}^{*}I^{c^{*}}(x,y)]\}$$

Where *I^c^*(*x*, *y*) is the input image, *R^c^*(*x*, *y*) is the reflected component of the input image, *c* is the colour channel and (*x*, *y*) is the pixel value. $R_{\mathrm{MSR}}^{c^{*}}(x,y)$ represents the output image of the improved MSR algorithm; *c* is the red, green and blue colour channels; *J*_*t* + 1_(*p*) is derived from Equation 10. The improved MSR algorithm not only effectively solves the problem of colour distortion after underwater image recovery, but also effectively suppresses the noise, protects the edges and enhances the details, not to mention that there is no colour bias problem.

#### 3.1.4 Contrast enhancement algorithm with multi-weight fusion

For the phenomenon of low contrast in underwater static blurred images, we make use of the improved contrast-constrained histogram equalisation (CLAHE) algorithm (Wang et al., 2025) on the basis of the *I*_MSR*_ of the image processed by the improved MSR algorithm. We introduce adaptive gamma correction into the CLAHE algorithm, and apply adaptive gamma correction to the H-space of the underwater image after CLAHE processing, in order to bring out the background details of the image and enhance the overall contrast of the image while improving the local contrast of the image, especially for the enhancement of the enhancement effect of the small difference between neighbouring regions. The adaptive gamma correction uses the compensated cumulative distribution function as an adaptive parameter to modify the intensity by the gradual increment of the original trend, gradually increasing the low intensity to avoid the significant attenuation of the high intensity, as shown in Equations 14–17.


(14) $$T(l)=l_{\max}{\left(\frac{l}{l_{\max}}\right)}^{1-{\mathrm{cdf}}_{w}(l)}$$

(15) $${\mathrm{cdf}}_{w}(l)=\sum_{l=0}^{l_{\max}}\frac{{\mathrm{pdf}}_{w}(l)}{\sum {\mathrm{pdf}}_{w}}$$

(16) $${\mathrm{pdf}}_{w}(l)=\mathrm{pd}f_{\max}{\left(\frac{\mathrm{pdf}(l)-\mathrm{pd}f_{\min}}{\mathrm{pd}f_{\max}-\mathrm{pd}f_{\min}}\right)}^{\alpha }$$

(17) $$\sum {\mathrm{pdf}}_{w}=\sum_{l=0}^{l_{\max}}{\mathrm{pdf}}_{w}(l)$$

Where *l_max_* in Equation 14 is the maximum intensity of the input, *cdf_w_*(*l*) is the cumulative distribution function; *cdf_w_*(*l*) in Equation 15 is the probability density function of the histogram after slight modification of the histogram using the weighted distribution function, *α* is the adjustment parameter, *pdf_max_* is the maximum probability density function value of the statistical histogram, *pdf_min_* is the minimum statistical histogram probability density function value of the statistical histogram.

We fuse the CLAHE algorithm with the adaptive gamma correction algorithm in the following processing steps:

(1) The image processed by the improved colour restoration algorithm based on MSR is partitioned into consecutive non-overlapping sub-blocks of size *M* × *N*, each containing n pixels. There is a close correlation between the size of these sub-blocks and the image contrast, the larger the sub-blocks are, the more obvious the contrast enhancement effect is, but at the same time, it will also lead to the loss of more detailed information.
(2) Distribution histogram analysis is performed on the sub-blocks of the underwater image and a threshold is set to excise the portion of the histogram that is above the threshold, and the excised portion is evenly distributed to the bottom of the histogram.
(3) By redistributing the histograms, histogram equalisation is performed on the new sub-block and the pixel positions of the regional blocks are performed to finally obtain the image *I_CLAHE_*.
(4) Perform adaptive gamma correction on image *I_CLAHE_*. Firstly, the image *I_CLAHE_* is converted to the HSV colour model, and the V-space in the HSV colour model is subjected to adaptive gamma correction, while the *H*, S-space colours are kept unchanged, and finally the image *I_ACG_* is obtained.

In order to solve the problem of unclear details of the image, finally the obtained image *I_ACG_* is fused with multiple weights, and we select contrast weights, brightness weights, and saliency weights to be fused to process the image. The contrast weights clearly show the edge feature information of the image; the luminance weights are responsible for assigning high values to pixels with good visibility, and this weight map is calculated by observing the deviation between the input *R*, *G*, and *B* channels and the luminance channel *L* (the average of the pixel intensities at a given location); the saliency weights, in order to highlight regions of the underwater image that have a higher degree of prominence.

The result can be obtained by subtracting the mean value of the input from its Gaussian smoothing. The three weight maps are combined into a normalised weight map, from which a 5-layer Gaussian pyramid is derived, as Gaussian pyramids are very effective in representing weights.

#### 3.1.5 De-dynamic fuzzification algorithm

**Figure 2:**
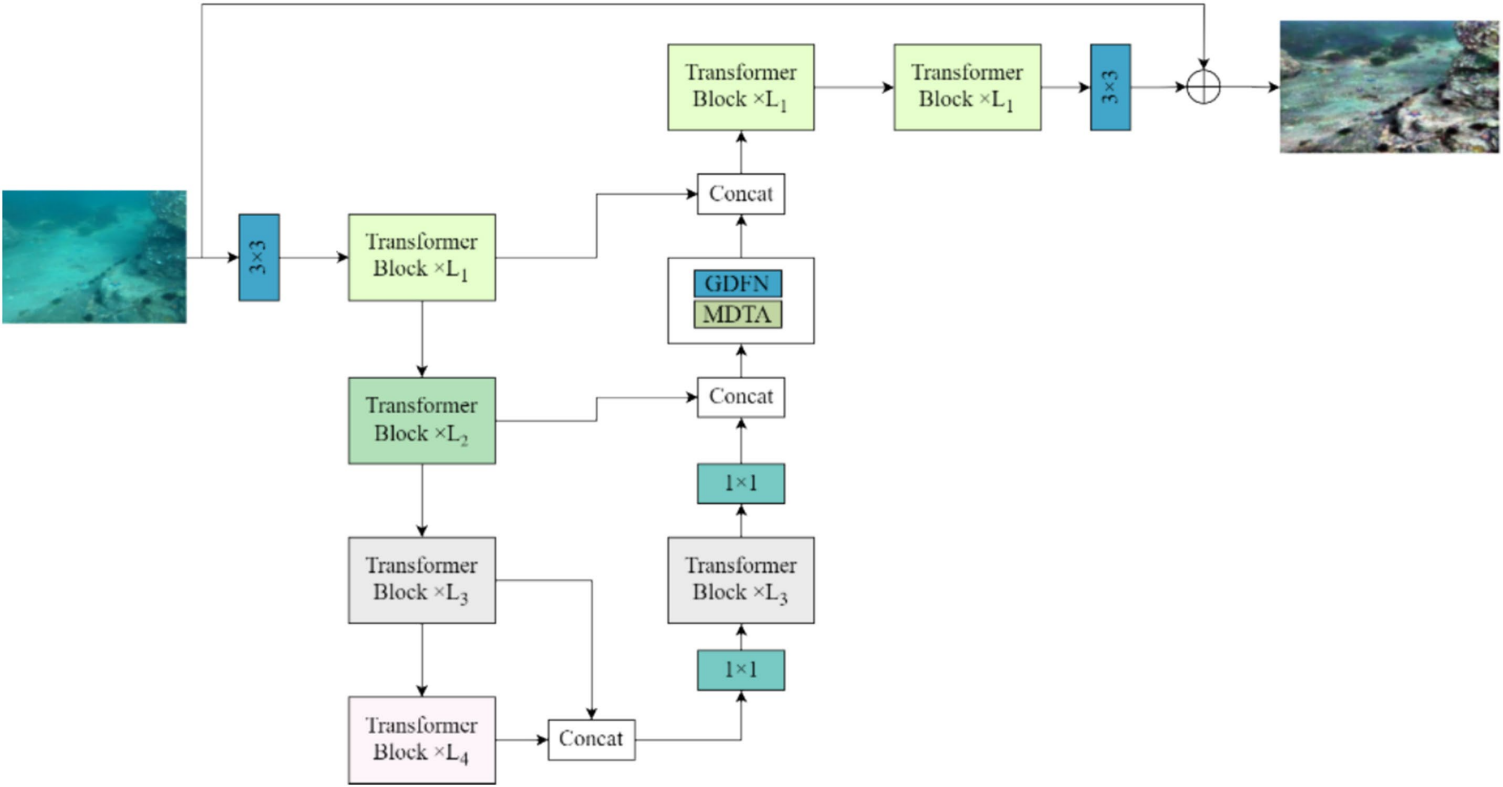
Restormer model overall structure diagram.

We use the Restormer network, an algorithm capable of removing dynamic blur from dynamic blurred images acquired from the air medium, to remove dynamic blur from underwater dynamic blurred images (Wang et al., 2024). The Restormer network is a computationally efficient encoder-decoder structured encoder-decoder converter for processing underwater images. Converter that learns multi-scale local and global aspects of high-resolution underwater images without decomposing them into local windows, thus alleviating computational bottlenecks by linking contextual features using remote images. The overall structure of Restormer network is shown in Figure 2.

As can be seen from Figure 2, for a given underwater motion blur image *I*∈*R*^*H* × *W* × 3^, the Restormer network model first applies a 3 × 3 convolution to obtain the low-level image features *F*_0_∈*R*^*H* × *W* × 3^ in the underwater motion blur image, and then these low-level image features in the underwater motion blur image are passed through the four-stage symmetric encoder. Decoder structure and transformed into deep underwater motion blur image features *F_d_*∈*R*^*H* × *W* × 3^. Moreover, each level of the encoder-decoder structure contains multiple Transformer modules, where the number of Transformer modules is gradually increased from top to bottom, thus maintaining the efficiency of extracting feature information from underwater images. Starting from the high resolution inputs the encoder is layered to reduce the space size while increasing the channel capacity. The decoder, on the other hand, takes potential features. $F_{l}\in R^{\frac{H}{8}\times \frac{W}{8}\times 8C}$ in low-resolution form as input and gradually recovers high-resolution underwater image features. For feature downsampling and upsampling in underwater dynamic blurred images, the model applies pixel cancellation and pixel disruption operations, respectively. To aid in the process of recovering clear underwater images, the features in the encoder are connected to the decoder features through jump connections. The linking operation is followed by a 1 × 1 convolution that is able to halve the number of channels at all levels. In the second step of the Restormer network model, it allows the Transformer module, the MDTA module and the GDFN module to aggregate the low-level image features of the encoder with the high-level image features of the decoder, an operation that facilitates the preservation of fine structural and textural details in the recovered image. Next, the deep features *F_d_* are further enriched in a high spatial resolution refinement stage. Finally, a convolutional layer is applied to the improved features to generate the residual image *R* ∈ *R*^*H* × *W* × 3^.

### 3.2 CFEC-YOLOv7 underwater target detection model

**Figure 3:**
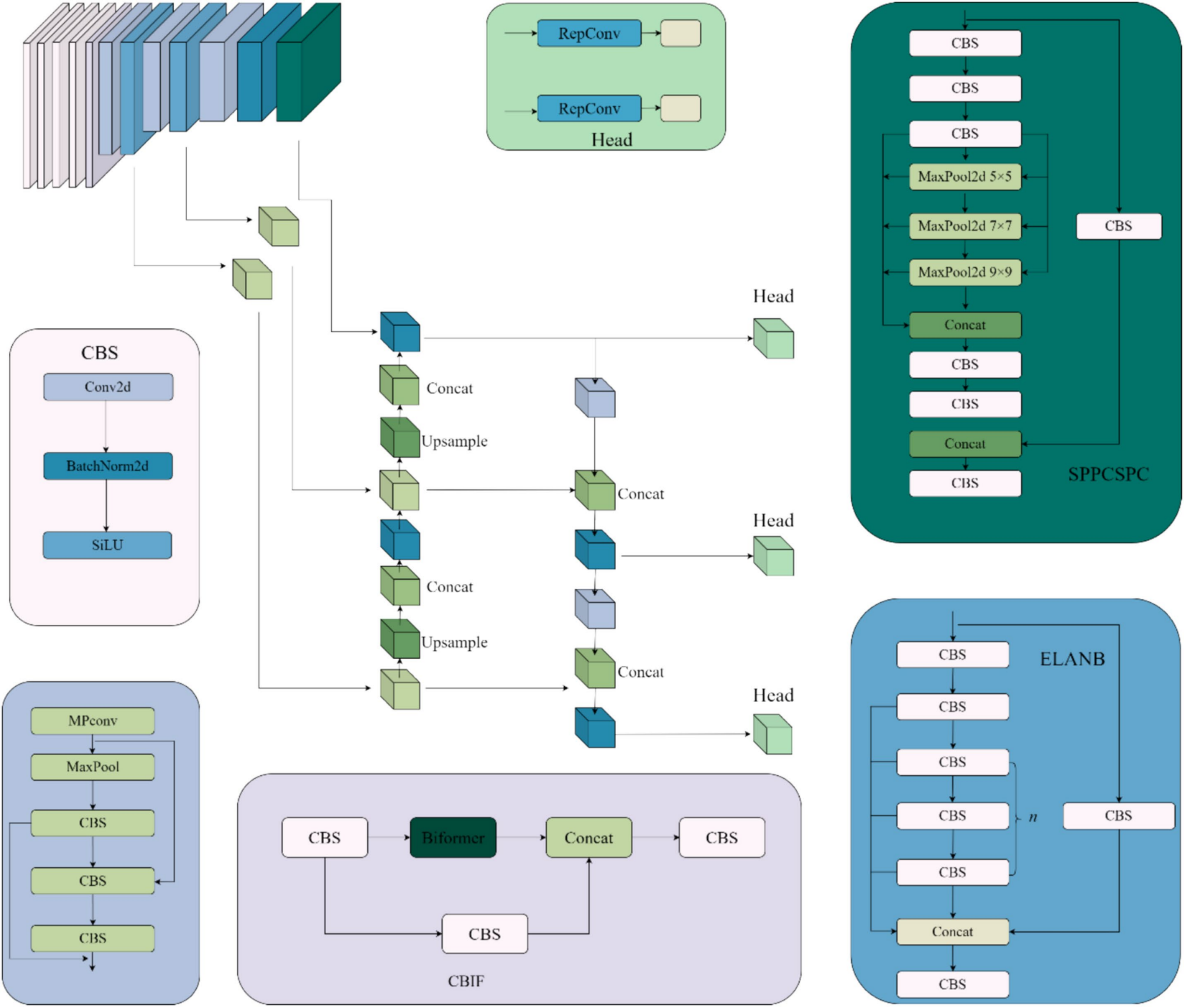
CFEC-YOLOv7 module structure diagram.

We propose an improved YOLOv7-based framework called CFEC-YOLOv7 for underwater target recognition. The network structure, shown in Figure 3, is designed to enhance both detection accuracy and speed. It introduces the CCBC module, which uses a self-attention mechanism to capture long-range dependencies, improves feature learning through global feature sampling, and boosts recognition performance. The original neck network, which includes ELANN and ELANB modules, is replaced with FasterNet to reduce computational cost and latency by using shortcut connections that promote feature reuse and efficient information interaction across channels. Additionally, we introduce the ECLOU loss function to accelerate bounding box regression, improve localization accuracy, and enhance model robustness. These improvements together make CFEC-YOLOv7 more effective and efficient for underwater object detection.

#### 3.2.1 FasterNet module and PConv operator

**Figure 4:**
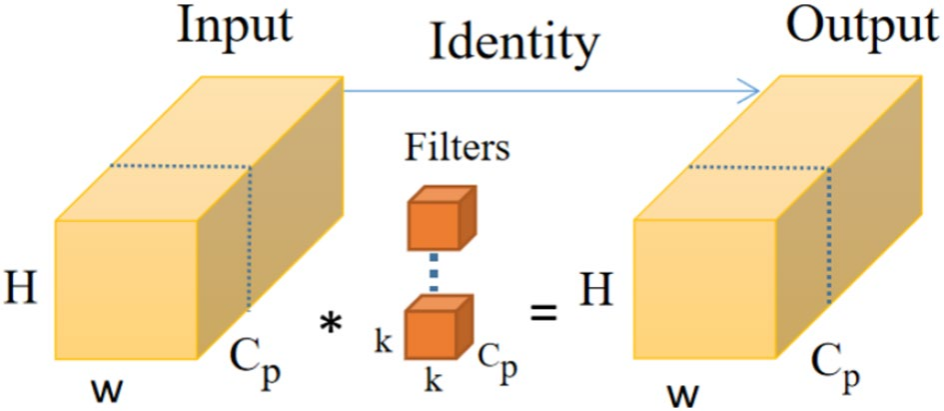
PConv operator structure diagram.

**Figure 5:**
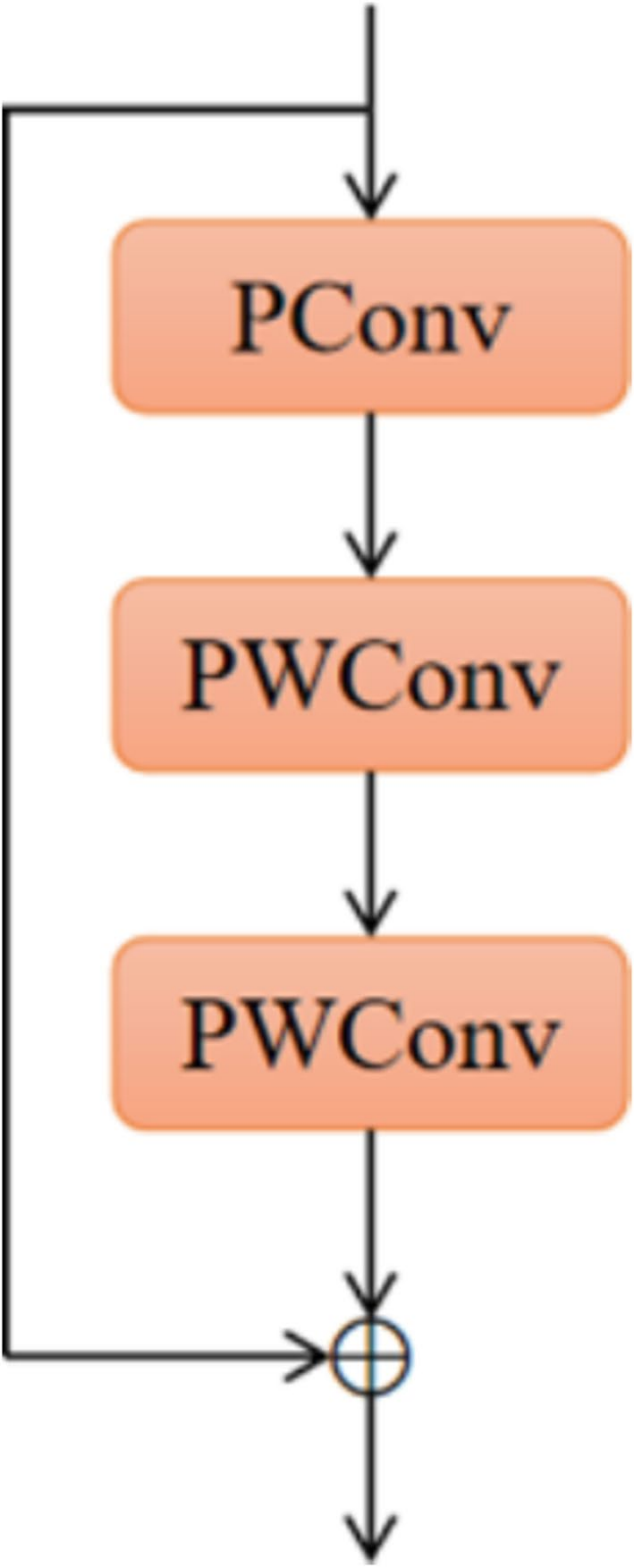
FasterNet module structure diagram.

As shown in Figure 4, the PConv (Zamir et al., 2022) operator takes advantage of the redundancy in feature mapping by systematically applying regular convolution to some of the information inputs of the features extracted from the previous convolution while keeping the rest of the channels unchanged, and this convolution is designed to reduce memory redundancy and the number of memory accesses, and the FasterNet module that utilises a collection of PConv convolution operators, as shown in Figure 5, to reduce the overall computational speed, which assembles the partial convolution operator (PConv) and two point-by-point convolution (PWConv) layers (Zhu et al., 2023), which together are presented as inverted residual blocks, where the middle layer has an extended number of channels and shortcut connections are placed to reuse input features. In addition, batch normalization (BN) is used to merge into neighbouring Conv layers, resulting in faster inference and reduced redundant computation and memory access.

Replacing the ELAN module in the neck network with the FasterNet module enables shortcut connections to reuse input features and enhances feature fusion, thus allowing the model network to reduce the number of memory redundancies and memory accesses and better utilise the computational power of the device, which not only reduces the number of parameters in the overall computation but also improves the speed of model recognition while enhancing the accuracy of the model.

#### 3.2.2 CCBC module

**Figure 6:**
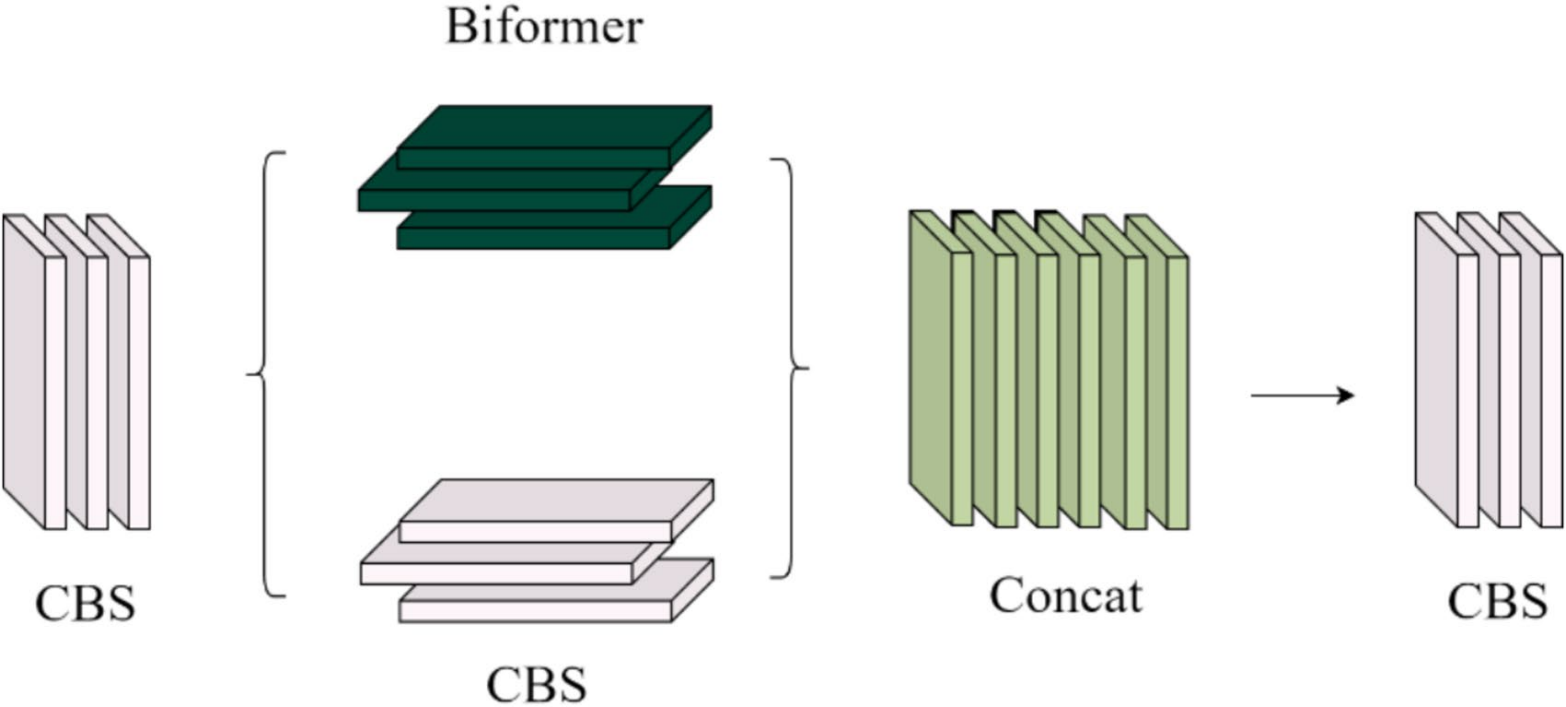
CCBC module structure diagram.

As shown in Figure 6, the CCBC module fuses local and global features in a parallel interactive way. It incorporates the Biformer module for global feature extraction, which uses a two-layer routing self-attention mechanism to adaptively sample the feature matrix and capture multi-scale global semantic information. This provides rich shallow semantic details for deeper convolutional layers. Local feature extraction is handled by the CBS module.

As shown in Figure 6, the CCBC module processes input features through two parallel branches. One branch uses the CBS and Biformer modules to extract local features, while the other performs global sampling of spatial features and captures global semantic information using a dual-layer routing self-attention mechanism. This mechanism adaptively queries features and models long-term dependencies, enhancing global feature representation. The outputs from both branches are summed and merged, effectively combining local and global information. This fusion enriches the semantic details for deeper convolutional layers and improves underwater target recognition accuracy by incorporating positional information.

**Figure 7:**
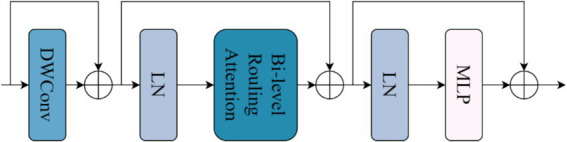
Biformer module structure diagram.

The structure of the Biformer module in Figure 6 is shown in Figure 7. First, the features extracted by the CBS module are embedded into the Biformer module, and then the feature information is globally sampled using the Biformer module (Reza, 2004), and the DWConv (Zamir et al., 2022) is used in the Biformer module to obtain the relative position information of the implicit encoding, and then, the BRA module and the MLP module with a 2-layer expansion ratio of e are used in turn to perform the cross-position relationship modelling and location-by-location embedding. Relationship modelling and position-by-position embedding, and then global sampling.

**Figure 8:**
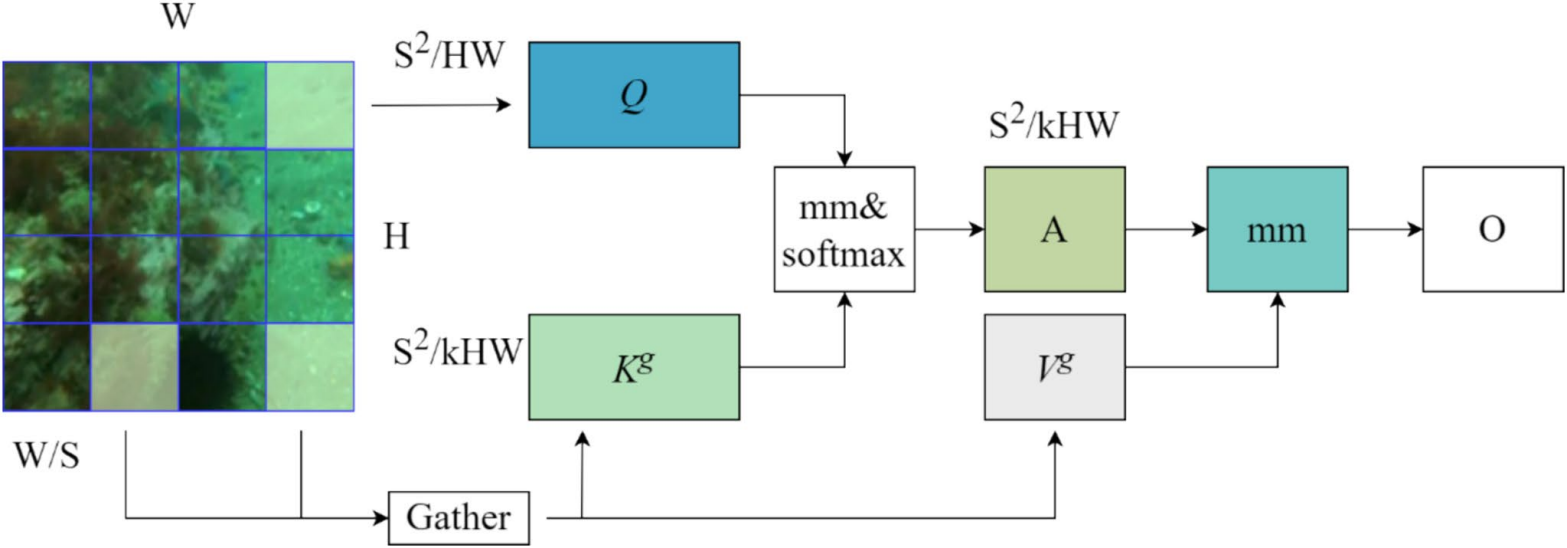
BRA Structure diagram.

The Bi-Level Routing Attention (BRA) mechanism in Figure 7 is the core part of the Biformer module, which is a dynamic query-aware sparse attention mechanism, as shown in Figure 8, that mainly handles a small fraction of relevant tokens in a query-adaptive manner without distracting other irrelevant tokens, for adaptive queries, irrelevant key-value pairs are firstly filtered out at the coarse region level so that only a maintains a small portion of the routing region, and then applies fine-grained token-to-token attention in the union of the remaining candidate regions (i.e., the routing region) with feature information that captures long-term dependencies for modelling, mainly by globally sampling image information features.

The BRA module on region partitioning and input projection, for an input feature map *X*∈*R*^*H* × *W* × *C*^, is first partitioned into *S* × *S* non-overlapping regions so each region contains *HW*/*S*^2^ feature vectors. This step is accomplished by reshaping *X* into and then deriving *Q*, *K*, and $V\in R^{S^{2}\times \mathrm{HW}/S^{2}\times C}$ , the linear projections. As shown in Equation 18.


(18) $$Q=X^{r}W^{q},\;\;\;\;K=X^{r}W^{k},\;\;\;\;V=X^{r}W^{v}$$

where *W^q^*, *W^k^*, *W^v^*∈R*^C × C^* are the projection weights of the query, key, and value, respectively.

Then, we find the participation relationship (i.e., the region in which each given region should participate) by constructing a directed graph. Specifically, we first derive region-level inquiries and keys, Q*^r^*, $K^{r}\in R^{S^{2}\times C}$ , by applying the average value of each region to *Q* and *K*, respectively. Then, the adjacency matrix $A^{r}\in R^{S^{2}}\times R^{S^{2}}$ of the region-to-region affinity graph is derived by matrix multiplication between *Q^r^* and the transposed *K^r^.* As shown in Equation 19.


(19) $$A^{r}=Q^{r}{\left(K^{r}\right)}^{T}$$

The entries in the adjacency matrix *A^r^* measure the degree to which two regions are semantically related. The next step in performing the core is to prune the association graph by retaining only top-k connections for each region. Specifically, a routing index matrix $I^{r}\in N^{S^{2}\times k}$ is derived using the operator. As shown in Equation 20.


(20) $$I^{r}=\text{topkIndex}\;(A^{r})$$

With the region-to-region routing index matrix *I^r^*, fine-grained token-to-token attention can be applied. For each query token in region *I*, it will be concerned to reside in $I_{\left(i,1\right)}^{r},I_{\left(i,2\right)}^{r},\ldots ,I_{\left(i,k\right)}^{r}$ . To cope with the possibility that these routing regions will be dispersed over the whole feature mapping, the key-value tensor is first collected, i.e. As shown in Equation 21.


(21) $$K^{g}=\text{gather}(K,I^{r}),V^{g}=\text{gather}(V,I^{r})$$

Where $K^{g},V^{g}\in R^{S^{2}\times \frac{\mathrm{kHW}}{S^{2}}\times C}$ . Attention can then be focussed on the collected key-value pairs. As shown in Equation 22:


(22) $$O=\text{Attention}(O,K^{g},V^{g})+\mathrm{LCE}(V)$$

Here, we will introduce a local context enhancement term *LCE*(*V*) as shown in Equation 22. The function *LCE*() uses a deep convolutional parameterization, where the kernel size is set to 5.

#### 3.2.3 ECLOU loss function

The accuracy of the network model localization is mainly dominated by the regression loss function, to have a higher accuracy we will propose a new enhanced loss function ECLOU as shown in Equation 23, which increases the prediction frame tuning and speeds up the frame regression rate, it is mainly based on two loss functions CLOU (Wang et al., 2022) and ELOU (Chen et al., 2023), the aspect ratio of the predicted frames are firstly altered by CLOU until it converges to a suitable range, and then each edge is carefully adjusted by ELOU until it converges to the correct value.


(23) $$\begin{array}{l} \;\text{ECIOULoss}=1-\mathrm{IOU}+\mathrm{\alpha v}\\ +\frac{\rho^{2}(b^{g}t,b)}{c^{2}}+\frac{\rho^{2}(h^{\mathrm{gt}},h)}{c_{h}^{2}}+\frac{\rho^{2}(w^{\mathrm{gt}},w)}{c_{w}^{2}} \end{array}$$

## 4 Experimental evaluation index and result analysis

### 4.1 Dataset preprocessing

We use the RUIE dataset to evaluate the effectiveness of our CFEC-YOLOv7 network model. This dataset contains 4,262 real underwater images collected from a marine environment platform, including three types of small targets—sea cucumbers, sea urchins, and starfish—in various water conditions. It reflects both the performance of image enhancement methods and our proposed model. For training and evaluation, we split the dataset into training, validation, and test sets in a 6:2:2 ratio, with all images resized to 400 × 300 pixels. We enhance all images using our proposed de-static blurring and de-dynamic blurring algorithms, transforming the original RUIE dataset into an enhanced version called RUIES, which is then used as input for our underwater target detection model.

### 4.2 Experimental evaluation index

The performance evaluation metrics of target recognition algorithms we use are Precision, Recall, and mean average precision (mAP).

#### 4.2.1 Precision and recall

Precision and recall are used as a measure of classifier precision in the field of deep learning, and the two are often incompatible relationship, in general, the higher the precision rate, the lower the recall rate, the formula for precision and recall are shown in Equations 24, 25. Precision rate is the proportion of correctly identified targets to the number of correctly identified and incorrectly identified targets. Recall is calculated by dividing the number of correctly identified targets by the sum of correctly identified targets and the number of undetected targets.


(24) $$\;\text{Precision}=\frac{\mathrm{TP}}{\mathrm{TP}+\mathrm{FP}}$$

(25) $$\text{Recall}=\frac{\mathrm{TP}}{\mathrm{TP}+\mathrm{FN}}$$

In Equations 24, 25, *TP* (True Positive) represents the sample predicted to be the target that is actually the target. *FN* (False Negative) represents the sample predicted to be the background but actually the target; *TN* (True Negative) is the sample that is accurately predicted to be the background that is actually the background: *FP* (False Positive) represents the sample that was predicted as the target but was actually the background.

#### 4.2.2 Mean average precision

Before introducing the mean average precision, it is necessary to have an understanding of the average precision (*AP*), which is defined as the average value of the precision rate under different recall rates, as shown in Equation 26.


(26) $$\mathrm{AP}=\sum_{i=1}^{n}P(i)\mathrm{\Delta r}(i)=\int_{0}^{1}P(r)\mathrm{dr}$$

From Equation 26 it can be seen that the meaning of *AP* can be understood as the area of the curve formed by precision and recall, while mean average precision represents the average of mean precision, which is the average precision of all categories of targets. *mAP* is shown in Equation 27.


(27) $$\mathrm{mAP}=\frac{\sum_{n=1}^{N}\mathrm{AP}(n)}{N}$$

Where *n* denotes a single category and *N* denotes the number of all categories. In the experiments, we use *mAP* evaluation metrics to quantitatively analyze the target recognition algorithms.

### 4.3 Experimental results and analysis

#### 4.3.1 Qualitative evaluation and quantitative evaluation

To verify the effectiveness of the underwater image enhancement algorithm we proposed, we conducted the verification from two aspects: qualitative evaluation (subjective visual perception) and quantitative evaluation.

##### 4.3.1.1 Qualitative evaluation

**Figure 9:**
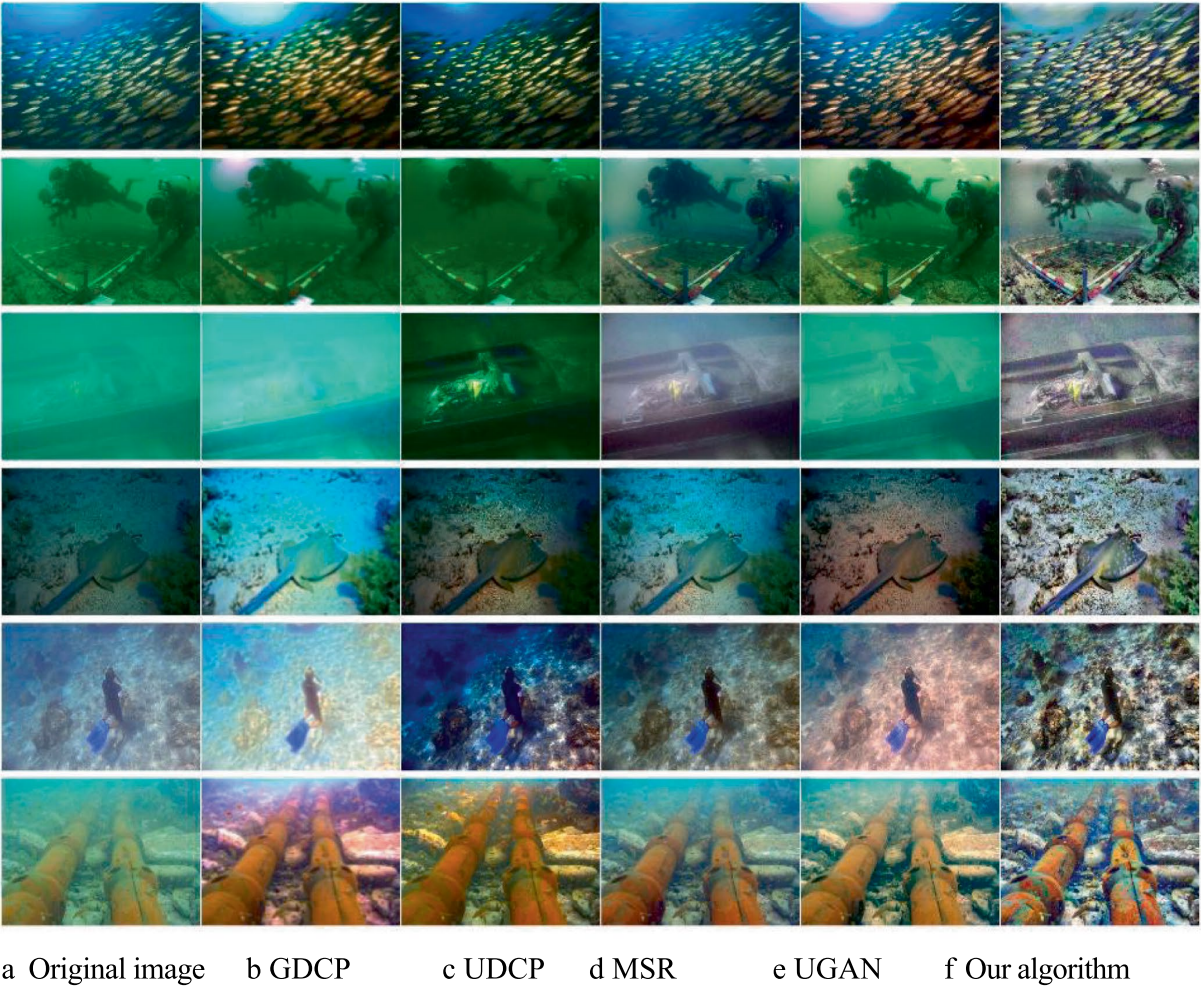
Qualitative evaluation experiment results compared with the effect diagram. **(a)** Original image; **(b)** GDCP; **(c)** UDCP; **(d)** MSR; **(e)** UGAN; **(f)** Our algorithm.

For the assessment of qualitative evaluation, we selected six representative underwater images, including underwater images with a blue background, underwater images with a green background, underwater images with low visibility, underwater images with weak light, underwater images with light scattering, and blurred images caused by particulate matter. For the six underwater images selected above, we conducted comparative experiments on the GDCP algorithm, the UDCP algorithm, the pre-improved MSR algorithm, and the UGAN network to verify the excellence of our image enhancement algorithm. The qualitative evaluation effect diagram is shown in Figure 9.

Figure 9 presents the qualitative evaluation of our algorithm and the existing methods. It can be seen from the figure that the brightness of the output image processed by the GDCP algorithm is overly saturated. The UDCP algorithm causes the phenomenon of low contrast and yellowish tone in the processed output image. Although the MSR algorithm corrects the color of the processed output image, problems such as excessive smoothness and color cast may occur. The UGAN network causes the problem of low contrast in the processed output image. The qualitative evaluation results of the algorithm we proposed are superior to the existing methods. It has a better visual effect than the existing methods. Meanwhile, this algorithm also has the characteristics of better naturalness, contrast, reduced color cast, maintained edge details, and no artifacts.

##### 4.3.1.2 Quantitative evaluation

Quantitative evaluation: Underwater Image Quality Metric (UIQM), Underwater Colour Image Quality Evaluation (UCIQE), information Entropy (Entropy), and average Gradient (AG) are selected as the evaluation criteria for the de-static blurring algorithm in this paper. Among them, a higher UIQM indicates that the underwater image quality based on human visual perception is better; a higher UCIQE indicates that the underwater image has better contrast, chromaticity and saturation. Entropy is mainly an objective evaluation index for measuring the amount of information contained in underwater images. The higher the Entropy, the richer the information content of the underwater image and the better the quality of the underwater image. AG mainly measures the clarity of underwater images. The higher the AG value, the higher the clarity of the underwater image, with better texture, contrast and edge clarity, and the better the quality of the underwater image.

In order to further enhance the effectiveness of the algorithm we proposed, it is verified by objective evaluation indicators. We calculated four objective evaluation indicators of underwater images, namely UCIQE, UIQM, AG and Entropy. The results of the quantitative evaluation experiment are shown in Table 1.

**Table 1:** Quantitative evaluation results.

Quantitative evaluation index	UCIQE	UIQM	AG	Entropy
Original image	0.39	0.11	6.03	6.86
GDCP	0.46	0.44	8.34	7.34
UDCP	0.45	0.17	7.92	6.91
MSR	0.44	0.25	6.61	7.07
UGAN	0.49	0.15	8.65	7.12
Our algorithm	0.52	0.47	15.29	7.63

It can be seen from Table 1 that for the evaluation index UCIQE, the underwater images processed by our proposed algorithm have a higher UCIQE value than those processed by other existing algorithms. This indicates that the underwater images processed by our proposed algorithm have better contrast, chromaticity and saturation. For the evaluation index UIQM, the UIQM value of underwater images processed by the algorithm proposed by us is higher than that of underwater images processed by other existing algorithms. This indicates that underwater images processed by the algorithm proposed by us have better visual perception for humans and better image quality. For the evaluation index AG, the AG value of the underwater images processed by the algorithm we proposed is nearly twice as high as that of the underwater images processed by other existing algorithms. This indicates that the processed underwater images have higher clarity, better texture, contrast and edge clarity, and better underwater image quality. For the evaluation index Entropy, the underwater images processed by the algorithm we proposed have a higher Entropy value than those processed by other existing algorithms. This indicates that the underwater images processed by the algorithm we proposed have more information and better underwater image quality.

Based on the comprehensive qualitative and quantitative evaluation results, the algorithm we proposed has a good enhancement effect on various types of underwater images, and the processed underwater images have better visual effects.

#### 4.3.2 Experimental results and analysis of underwater image enhancement

**Figure 10:**
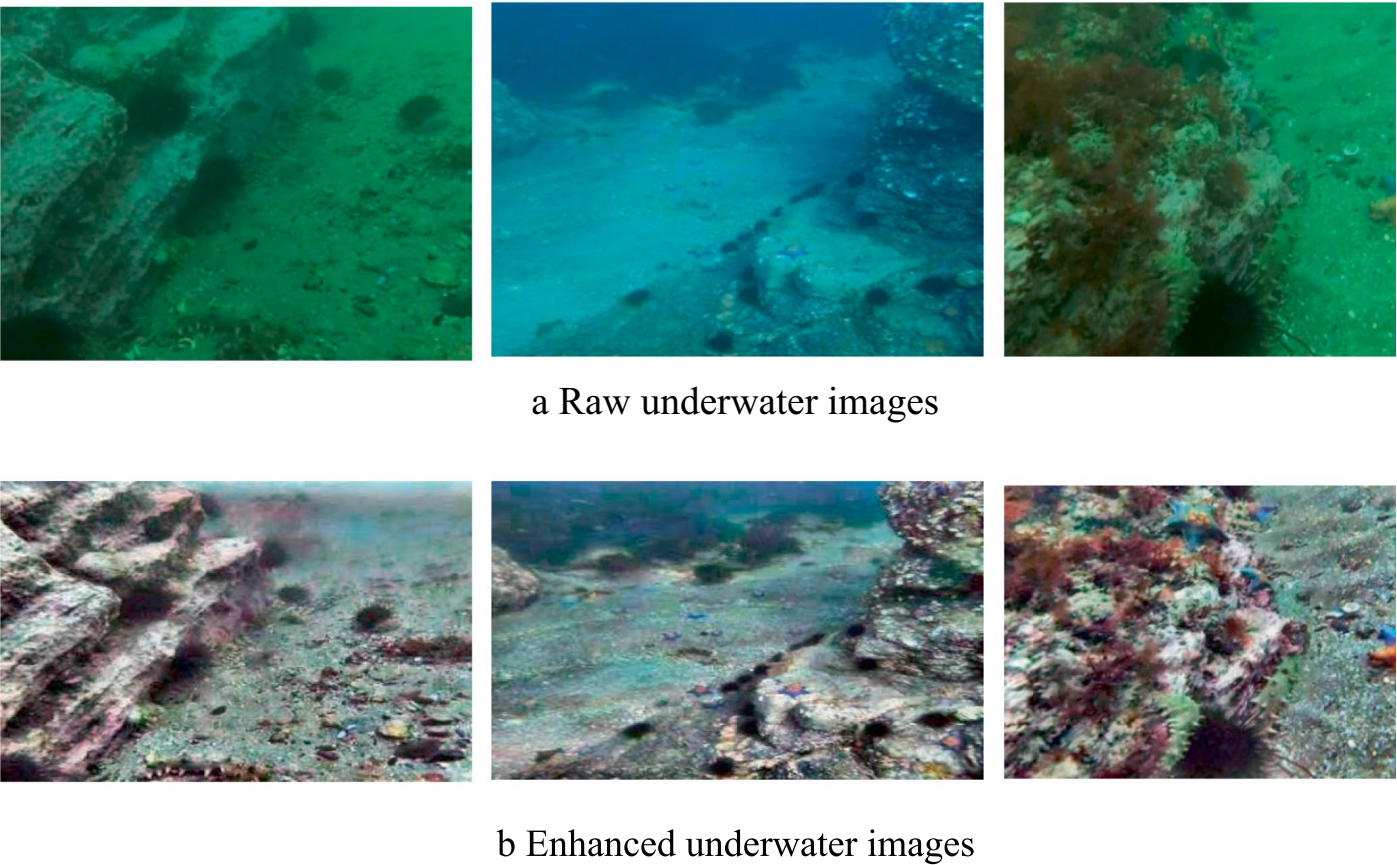
Underwater image enhancement before and after the effect comparison. **(a)** Raw underwater image; **(b)** Enhanced underwater images.

The effect graph after enhancement by our proposed underwater image enhancement algorithm is shown in Figure 10. Figure 10a represents the three original images before underwater image enhancement, and Figure 10b represents the effect diagram of the three original images after enhancement. As can be seen from the effect diagram, the enhanced underwater images have higher contrast, higher saturation, and richer and more realistic colours, solving the problems of static blurring such as green and blue bias as well as dynamic blurring in the original underwater images, and enhancing the brightness, saturation and contrast of the underwater images.

To investigate the effect of underwater image enhancement algorithms on the recognition accuracy of underwater dense and small-sized targets, we used the unimproved YOLOv7 network to train and test the original dataset RUIE dataset and RUIES dataset respectively, and the experimental results are shown in Table 2. In the original RUIE dataset, the Precision, Recall and average accuracy *mAP* of the network model are lower than those of the network model on the RUIES dataset, which indicates that in the original RUIE dataset, a lot of key information in the underwater image is not successfully extracted, and thus a lot of underwater targets are not accurately identified. Our proposed underwater image enhancement algorithm effectively solves this problem, restores the real and effective information of the targets in the underwater images, and improves the recognition accuracy of the network model for underwater targets.

**Table 2:** Comparison of experimental results before and after underwater image enhancement algorithm processing.

Model	Training sets	Verification sets	Testing sets	Precision (%)	Recall (%)	*mAP*@0.5
YOLOv7	RUIE dataset			84	83.6	84.1
YOLOv7	RUIES dataset			85.3	84.8	88.5

**Figure 11:**
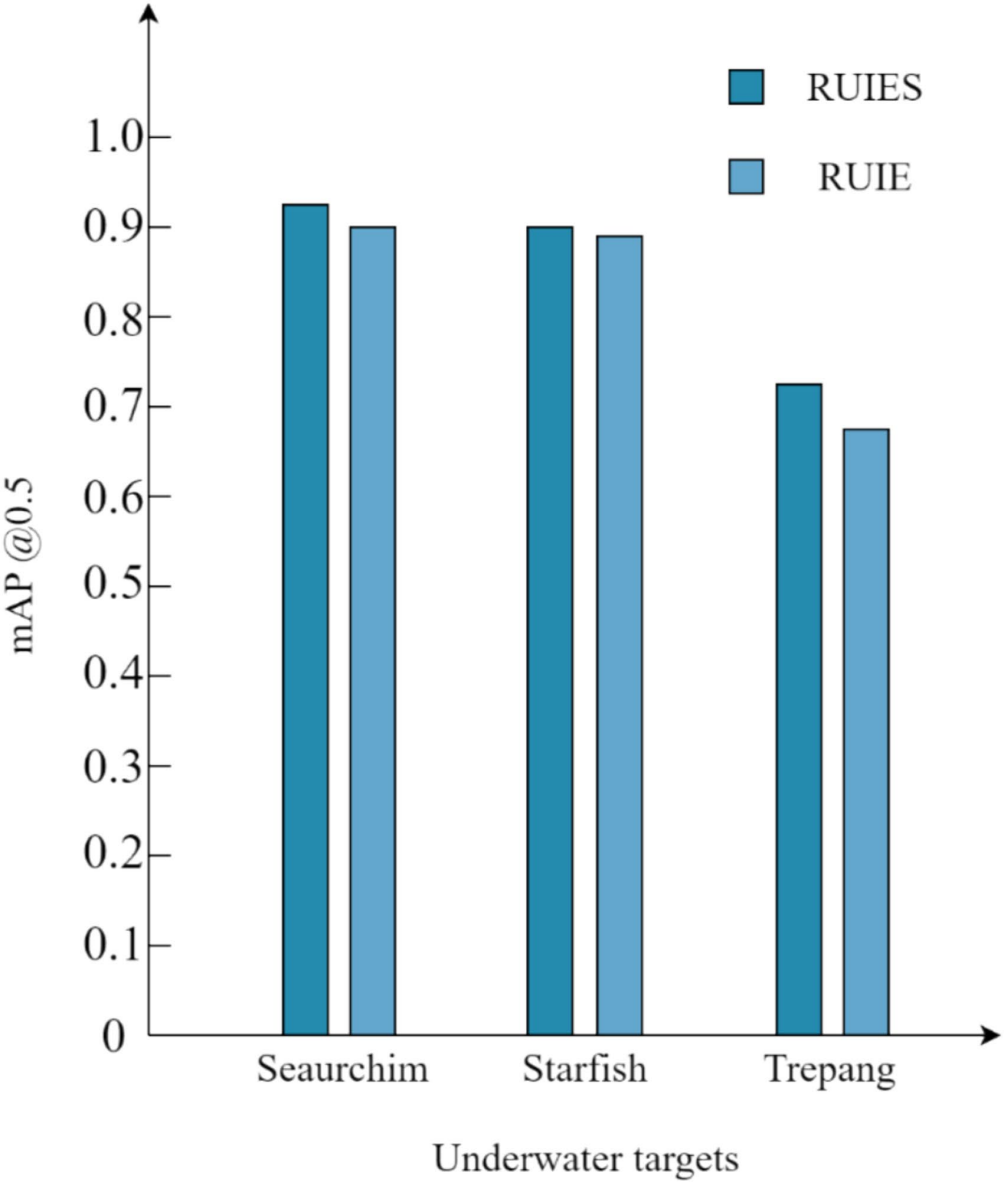
Comparison of *mAP* values of various objects before and after enhancement of the RUIE dataset.

As shown in Figure 11, the RUIES dataset processed by our proposed underwater image enhancement algorithm is compared with the unenhanced RUIE dataset, and the recognition accuracy of sea urchin and starfish in the RUIES dataset is slightly improved, and the recognition accuracy of sea cucumber is greatly improved, which indicates that our proposed underwater image enhancement algorithm is effective for the recognition of underwater targets.

**Figure 12:**
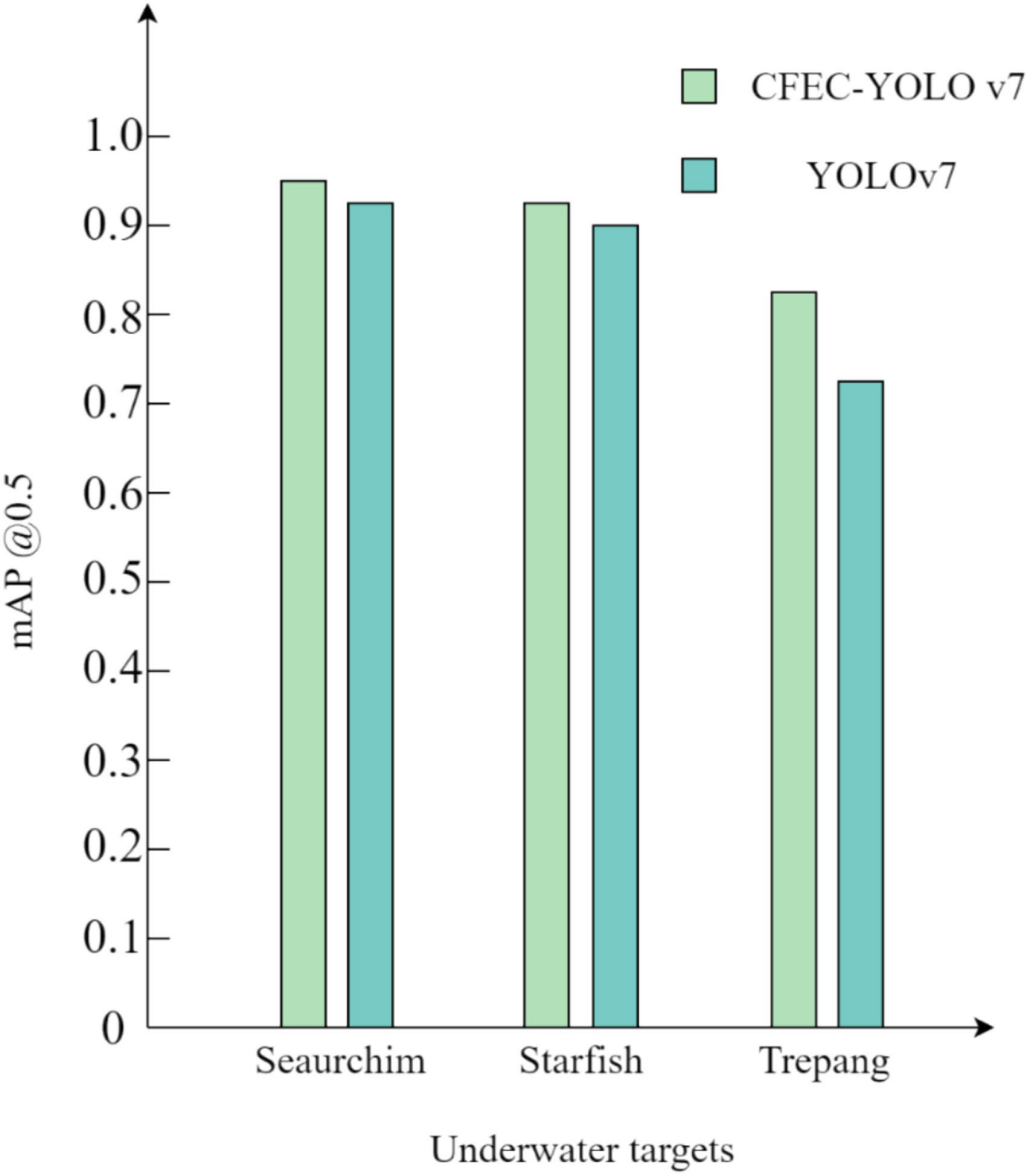
Comparison of mAP values before and after YOLOv7 improvement.

**Figure 13:**
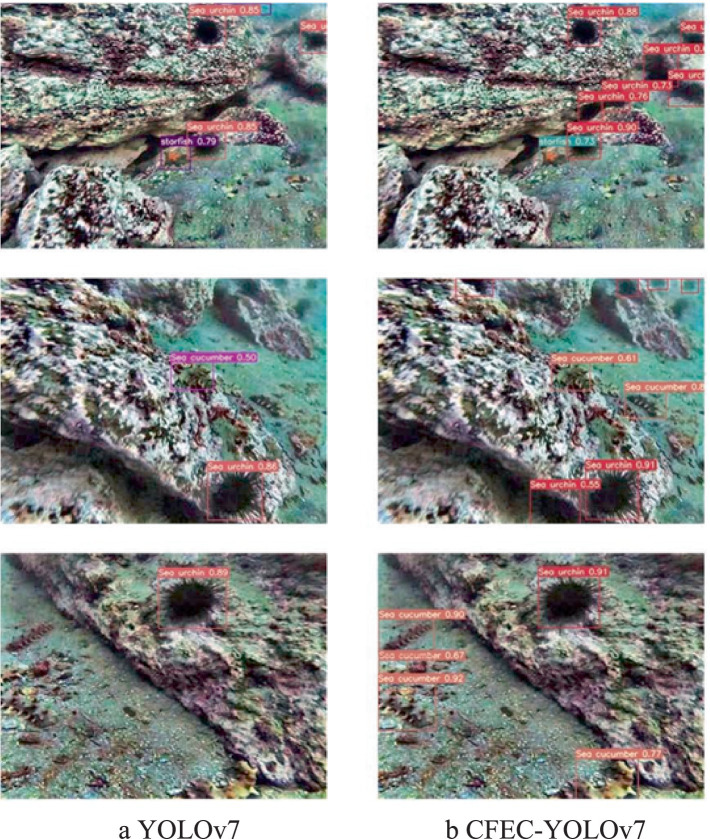
Comparison of recognition results between YOLOv7 network and YOLOV7-CBF network. **(a)** YOLOv7; **(b)** CFEC-YOLOv7.

To validate the effectiveness of our proposed CFEC-YOLOv7 network for underwater target recognition, the validation is performed on the RUIES dataset. The experimental comparison results of the CFEC-YOLOv7 network with the YOLOv7 network are shown in Table 3, the results of the *mAP* comparison of various types of targets are shown in Figure 12, and the graphs of the experimental visualization results are shown in Figure 13. Among them, Figure 13a shows the results of the original YOLOv7 network for underwater target recognition, Figure 13b shows the results of the improved CFEC-YOLOv7 network based on the YOLOv7 network for underwater target recognition.

**Table 3:** Comparison of experimental results before and after network model improvement.

Model	GFLOPs	Params (M)	Speed (fps)	Precision (%)	Recall (%)	*mAP*@0.5
YOLOv7	107.2	37.21	151	85.3	84.8	88.5
CFEC-YOLOv7	101.6	31.63	162	88.3	85.2	90.4

As shown in Table 3, the CFEC-YOLOv7 network outperforms the original YOLOv7 network in terms of accuracy Precision, recall, recognition accuracy *mAP*, and recognition speed for underwater dense and small-sized targets, with 3% improvement in Precision, 0.4% in Recall, 1.9% in *mAP* and 8.6% in recognition speed. Speed is improved by 8.6%. From Figure 13, it can be seen that the improved CFEC-YOLOv7 network has improved the recognition accuracy of sea urchins, starfish, and sea cucumbers compared with the pre-improved YOLOv7 network. Moreover, in Figure 13, it can be seen that the original YOLOv7 network has missed detection for dense and small-sized underwater targets, while our proposed CFEC-YOLOv7 network reduces the problem of missed detection of the original network. In conclusion, the experimental results show that the recognition performance of our proposed CFEC-YOLOv7 network for underwater dense and small-sized targets is better than the original network YOLOv7 in all aspects, which improves the recognition accuracy and speed of underwater targets.

#### 4.3.3 Ablation experiments

To evaluate the effectiveness of each module, we do a series of ablation experiments on the same experimental environment and dataset. The experimental results are shown in Table 4. Precision, recall, and *mAP* are the performance metrics to evaluate the accuracy. GFLOPs and Parameters denote the amount of model computation and the number of parameters, respectively.

**Table 4:** Performance comparison of various improvement strategies.

Number	FasterNet	CCBC	GFLOPS	Params (M)	Precision (%)	Recall (%)	*mAP*@0.5 (%)	Speed (fps)
1	–	–	107.2	37.21	151	85.3	84.8	88.5
2	√		38.0	31.20	81.4	82.8	85.6	188
3		√	103.2	34.08	88.2	83.8	89.9	136
4	**√**	**√**	101.6	31.63	88.3	85.2	90.4	162

As shown in Table 4, adding the CCBC module slightly increases the number of parameters and slightly reduces recognition speed due to the computational cost of the two-layer routing self-attention mechanism. However, it improves recognition accuracy by 1.4% in terms of *mAP*. Introducing the FasterNet module reduces both the number of parameters and computational load through point-wise convolution, significantly improving recognition speed and better utilising GPU performance. When both the CCBC and FasterNet modules are integrated into the original network, the overall performance is notably enhanced. In particular, the CCBC module in the backbone brings the most significant accuracy improvement by enhancing fealture extraction, while the FasterNet module in the neck greatly boosts inference speed.

#### 4.3.4 Comparison experiments

Our proposed CFEC-YOLOv7 network is compared with the other four detection networks for experiments on the RUIES dataset, and the training parameters of these four different network models and the training environment are consistent with ours, as shown in Table 5, in which the U-YOLOv7 network proposed in the literature (Yu et al., 2023) is built on top of YOLOv7 by combining the cross-transformation and the efficient squeezing excitation module and a decoupling head based on hybrid convolution is designed to increase the extraction of image information, which in turn improves the recognition accuracy, and the remaining three recognition networks, YOLOv5 (Sada et al., 2019), YOLOX (Ren et al., 2024), and YOLOv7-Tiny (Liu and Ye, 2023), are all existing network models.

**Table 5:** Experimental comparison diagram of different model algorithms.

Model	GFLOPs	Params (M)	Speed (fps)	Precision (%)	Recall (%)	*mAP*@0.5
YOLOv5	16.3	7.07	95	85.9	79.9	84.3
YOLOX	5.6	8.9	60	79.1	75.9	80.1
YOLOv7-Tiny	13.1	6.02	188	84.1	82.2	86.8
CFEC-YOLOv7	101.6	31.63	162	88.3	85.2	90.4
U-YOLOv7	34.2	10.91	135	82.6	87.8	86.2

As can be seen in Table 5, our proposed CFEC-YOLOv7 network model has the highest *mAP* compared to the other network models and also ranks second in terms of speed. As shown in Table 5, YOLOv7-Tiny has the highest recognition speed, but the recognition accuracy is significantly lower than that of our proposed CFEC-YOLOv7 model. U-YOLOv7 is an improved model based on YOLOv7, and as can be seen in Table 5, the number of model parameters and the amount of computation is much lower than that of our proposed model, and the overall performance is excellent, but its recognition accuracy and recognition speed are both However, its recognition accuracy and recognition speed are lower than that of our proposed improved network model. Although YOLOv5 is the most widely used network, its recognition accuracy and speed still need to be improved compared with our proposed network, and the difference between the YOLOX network and other networks is that it uses the Anchor free method when extracting target frames, and its recognition accuracy and speed are much lower than that of our proposed CFEC-YOLOv7 network. Network. The experimental results show that the speed and accuracy of our proposed CFEC-YOLOv7 model are well-balanced.

**Figure 14:**
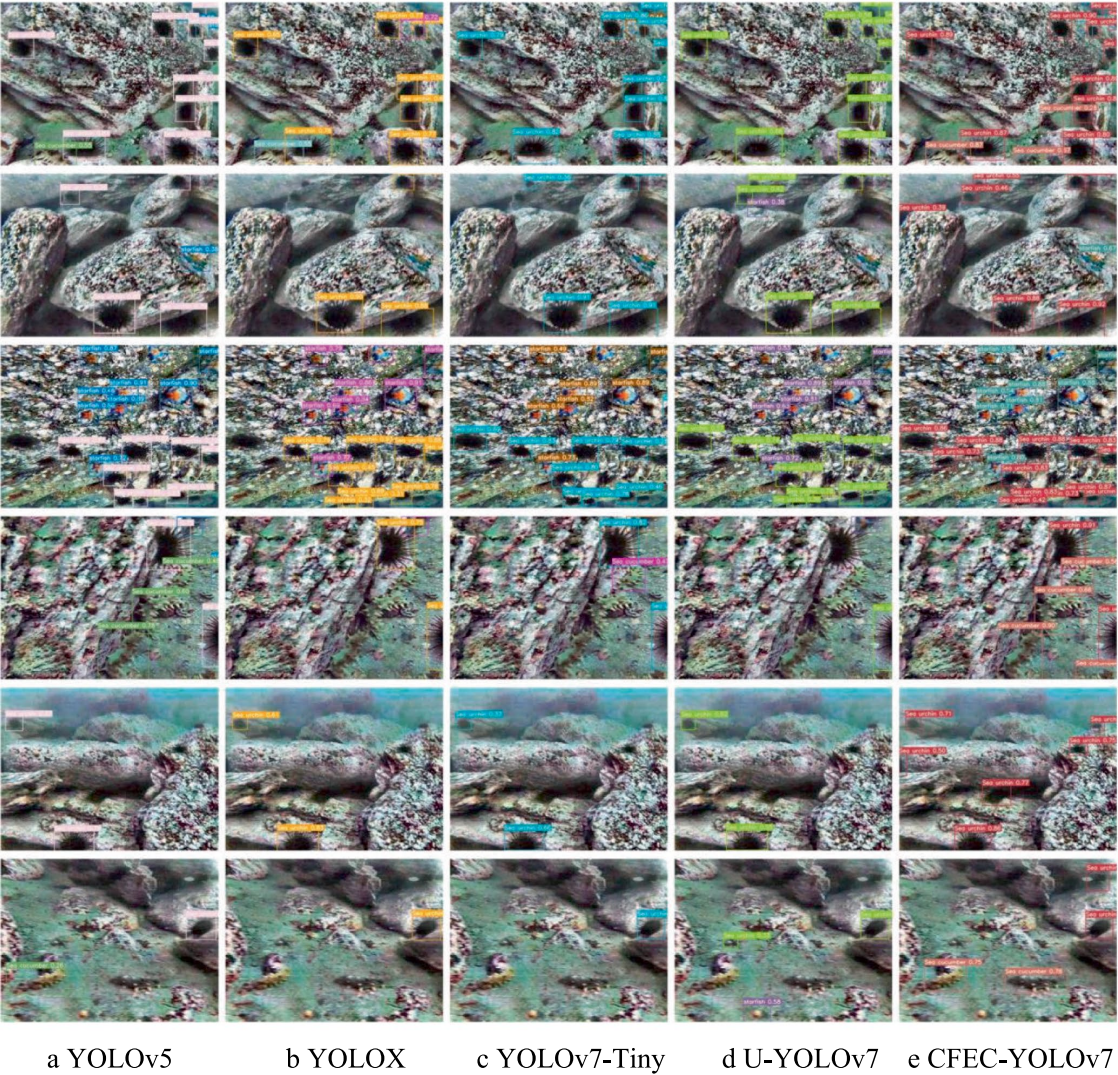
The proposed method is compared with the existing method. **(a)** YOLOv5; **(b)** YOLOX; **(c)** YOLOv7-Tiny; **(d)** U-YOLOv7; **(e)** CFEC-YOLOv7.

The results of comparing our proposed model with the existing models are shown in Figure 14. Observing the first set of graphs in Figure 14, the YOLOv5 network only has missed the detection of sea cucumbers, the YOLOX network has missed the detection of sea cucumbers for both sea cucumbers and sea urchins, and the YOLOv7-Tiny network and the U-YOLOv7 algorithm do not identify sea cucumbers at all, but the network designed by us can identify all the categories are recognised and there are no miss-detections.

Looking at the second set of plots in Figure 14, the YOLOv5 network, YOLOX network, YOLOv7-Tiny network, and the U-YOLOv7 algorithm all fail to identify sea urchins and starfish, while our proposed network only fails to identify starfish. Observing the third set of graphs in Figure 14, it can be seen that the targets in the third graph are denser, then the dense targets in the third set of graphs have higher requirements for the recognition network, in which the YOLOv5 network, the YOLOX network, the YOLOv7-Tiny network, and the U-YOLOv7 algorithm all have sea urchin miss-detections, and our designed CFEC-YOLOv7 network can identify all the categories without any miss-detection. Observe the fourth set of plots in Figure 14, where all other existing methods have missed sea cucumbers. Observing the fifth set of graphs in Figure 14, only our designed method recognises all the targets in all the categories in the image, whereas all the other existing methods have missed the detection of sea urchins. Observing the last set of graphs in Figure 14, all the existing methods showed missed detection of sea cucumbers and the U-YOLOv7 algorithm also showed wrong detection compared to the existing methods.

The experimental result graphs show that the CFEC-YOLOv7 network we designed reduces the leakage or misdetection that occurs during the recognition of underwater targets and has a higher recognition accuracy.

## 5 Conclusion and outlooks for future work

### 5.1 Conclusion

To solve the problem of static blurring in underwater images, we propose an adaptive color compensation algorithm, an improved color restoration algorithm based on msr, and a multi-weight fusion contrast enhancement algorithm for sequential processing of underwater static blurred images. Aiming at the problem of dynamic blur in underwater images, the dynamic blur removal weights trained by the Restormer network model are utilised to remove the dynamic blur in underwater dynamic blur images. To solve the problems of low recognition accuracy and slow recognition speed in complex underwater environments, we propose the CFEC-YOLOv7 network. We introduce the CCBC module we proposed into the backbone network of the CFEC-YOLOv7 network to fuse local and global feature information in a parallel interactive manner and obtain multi-scale global semantic information in the image. And provide rich shallow image semantic information for advanced deep convolutional features. Furthermore, the FasterNet module is introduced in the backbone network and the neck network, enabling quick connections to reuse the input features. Thus, the model network can reduce memory redundancy and the number of memory accesses, and make better use of the computing power of the devices. To further improve the accuracy of model positioning and enhance the robustness of the model, we also propose a new ECLOU loss function. The experimental results show that the underwater target recognition network proposed in this paper improves the recognition accuracy and speed of underwater targets.

### 5.2 Outlooks for future work

Due to the numerous factors causing dynamic blurring in underwater images, it is relatively difficult to completely remove the dynamic blurring in underwater images. Therefore, in future research, an attempt can be made to design a de-dynamic blurring algorithm that can completely remove the dynamic blurring in underwater images, and combine the underwater static blurring algorithm and the de-dynamic blurring algorithm to obtain underwater images of higher quality. For the research on underwater target recognition algorithms, the target recognition algorithm we proposed may not meet the requirements of future mobile devices. Therefore, in future research, we can attempt to design a more advanced, lightweight and mature underwater target recognition algorithm to achieve better application prospects.

## Data Availability

Publicly available datasets were analyzed in this study. This data can be found here: https://doi.org/10.48550/arXiv.1901.05320, https://github.com/xinzhichao/Underwater_Datasets/blob/main/README_zh-CN.md.
